# Supplementary material for: Evaluation of the Cytotoxic Effect of Pd2Spm against Prostate Cancer through Vibrational Microspectroscopies
Source: Int J Mol Sci. 2023 Jan 18;24(3):1888. doi: 10.3390/ijms24031888 (PMC9916163; doi:10.3390/ijms24031888)
Supplement: Supplementary file 1 [file ijms-24-01888-s001.zip › ijms-2147404-supplementary.pdf]

Evaluation of the Cytotoxic Effect of Pd_2Spm Against Prostate Cancer Through Vibrational Microspectroscopies

Raquel C. Laginha [1], Clara B. Martins [1], Ana L. Brandão [1], Joana Marques [1], M. Paula M. Marques [1,2], Luís A.E. Batista de Carvalho [1]*, Inês P. Santos [1,†] and Ana L.M. Batista de Carvalho [1,†]

[1] Molecular Physical-Chemistry R&D Unit, Department of Chemistry, University of Coimbra, 3004-535 Coimbra, Portugal

[2] Department of Life Sciences, Faculty of Science and Technology, University of Coimbra, 3000-456 Coimbra, Portugal

Supplementary material

***** Correspondence: labc@ci.uc.pt; Tel.: +351 239 854 462

† These authors contributed equally to this work

Table S1: Raman and infrared bands for human healthy and cancer prostate cells (PNT-2, LNCaP and PC-3). Features from specific drug-prompted DNA and protein conformational rearrangements are presented in red. The signals exclusively detected by infrared are shaded in grey.

Bands (cm^{-1})	[a]Assignment			
	Nucleic acids	Proteins	Lipids	Carbohydrates
1716-1701	A-DNA (ν(C=O))			
1688-1687		ν(C=O)$_{amino\ acid\ side\ chain}$		
1691-1669		Amide I/β-sheet, antiparallel		
1660-1648		Amide I/random coil		
1639		Amide I/β-sheet, parallel		
1659-1653	DNA (δ(NH))	Amide I (ν(C=O))/α-helix	ν(C=C)	
1617	A (ν(CC)$_{ring}$), C (δ(NH_2))	Phe, Tyr, Trp (ν(C=C)), δ(NH_2)		
1605	T (ν(CC)$_{ring}$)	Phe (δ(C=CH))		
1586-1582		ν(C=C), ν(C=N)	ν(C=C), ν(C=N)	
1577-1575	A,G (ν(CC)$_{ring}$)			
1555	G (ν(CC)$_{ring}$)	Trp (ν(CC)$_{ring}$), ν(C=C)$_{porphyrin}$		
1545		Amide II ((δ(CN-H)/ν(CN))		
1521		ν(C=C)$_{porphyrin}$		
1467-1465		δ(CH_2), δ(CH_3)	δ(CH_2), δ(CH_3), aromatic lipids	δ(CH_2)
1455-1442			δ(CH_2)	
1416	A-DNA (δ(CH_2))			
1398-1395		δ(CH_2), ϱ(CH_2)	membrane lipids (δ(CH_2))	δ(CH_2), ϱ(CH_2)
1379	A,G,T (ν(CC)$_{ring}$)	glycoproteins (δ(CH_3))	lipids/acyl chains (δ(CH_3))	saccharides (δ(CH_2))
1340-1339	G (ν(CC)$_{ring}$)			
1314-1309	G (ν(CC)$_{ring}$)	δ(CH_2)	δ(CH_2)	δ(CH_2)
1306-1303	RNA/A,C (ν(CC)$_{ring}$)			
1280-1279		amide III/α-helix	δ(CH_2), ω(CH_2), t(CH_2)	δ(CH_2), ω(CH_2), t(CH_2)
1260-1251	A,T (ν(CC)$_{ring}$)	δ(CH_2), δ(C=C–H)	δ(C=C-H)$_{phospholipids}$	δ(CH_2), ω(CH_2), t(CH_2)
1252-1240		amide III/random coil		
1238	B-DNA (ν$_{as}$(PO_2^-))			
1230-1225		amide III/β-sheet		
1221-1220		amide III (δ(NH), ν(CN))		
1209		Hyp, Phe, Tyr (ν(CC))		
1175-1167	C,G,T (ν(CC)$_{ring}$)	Tyr, Phe (δ(CH))		
1158-1157		ν(CC), ν(CN), δ(CH_2)	δ(CH_2), ν(=C–C=)$_{conjugated}$	δ(CH_2)
1129-1126	RNA/ribose (ν(CO))	ν(CN)	ν(CC)$_{acyl}$ (*trans* conformation)	ν(CO), ν(CC)
1096-1091	B-DNA (ν$_s$(PO_2^-))			
1083-1077		ν(CC), ν(CN)	phospholipids (ν$_s$(PO_2^-))	glycogen (ν(CC), ν(CO))
1063-1058	B-DNA/deoxyribose (ν(CO))	ν(CC), ν(CN)	ν(CC), ν(CO)	ν(CC), ν(CO), δ(OCH)
1051-1050	RNA (ν(OPO))		phosphate esters (ν$_{as}$(OPO))	ν(C–OH)
1035-1031		Phe (δ(CH)), ν(O–CH_3)	ν(CC), phospholipids (δ(CH))	ν(CC), ν(CO), ν(C–OH)
1004		Phe (ν$_s$(CC)$_{ring}$)		
980		ν(CC) (β-sheet)	δ(C=CH)	
962-961	DNA (ν(CC)/ν(CO)$_{backbone}$)			polysaccharides (δ(C=O))
945-944	RNA/ribose (ν(CC)$_{ring}$)			polysaccharides (skeletal modes)
937-935		ν(CC) (α-helix)	glycolipids (ν(COC))	ν(COC)$_{glycosidic}$
903-897	deoxyribose (ν(CC)$_{ring}$)	ν(CC)	fatty acids (ν(CC), ν(CO))	ν(COC)
884-883		ϱ(CH_2)		
855-854		Pro, Tyr, Val (ν(CC), δ(CCH))		polysaccharides (ϒ(COC))
833-831	B-DNA (ν(OPO)$_{backbone}$)	Pro, Tyr (ν(CC))		
816-813	RNA (ν(OPO)$_{backbone}$)	Pro, Tyr (ν(CC))		
787-784	C,T,U (ν(CC)$_{ring}$)			
753-750	Z-DNA (ν(OPO)$_{backbone}$)			
752-750	B-DNA/dT (ν(CC)$_{ring}$),	Trp (ν$_s$(CC)$_{ring}$)		
731-726	B-DNA/A (ν(CC)$_{ring}$)	Trp (ν$_s$(CC)$_{ring}$)		
679-677	B-DNA/A,G,T,C (ν(CC)$_{ring}$)	Trp (ν(CC)$_{ring}$)		
670	A-DNA/dG (ν(CC)$_{ring}$)			
668	B-DNA/G,T (ν(CC)$_{ring}$)			
645-644		ν(CS), Tyr (t(CC))		
626-623	Z-DNA/dG (ν(CC)$_{ring}$)			
624-623		Phe (t(CC))		

[a]A – adenine; C – cytosine; dG – deoxyguanine; dT – deoxythymine; G – guanine; Glu – glucose; Hyp – hydroxyproline; Met – methionine; Phe – phenylalanine; Pro – proline; T – thymine; Trp – tryptophan; Tyr – tyrosine; U – uracil; Val – valine. δ – in-plane deformation; ϒ – out-of-plane deformation; ν – stretching; ϱ – rocking; t – twisting; ω – wagging. s – symmetric; as – anti-symmetric.
